# Novel Online Dimensionality Reduction Method with Improved Topology Representing and Radial Basis Function Networks

**DOI:** 10.1371/journal.pone.0131631

**Published:** 2015-07-10

**Authors:** Shengqiao Ni, Jiancheng Lv, Zhehao Cheng, Mao Li

**Affiliations:** Machine Intelligence Laboratory, College of Computer Science, Sichuan University, Chengdu 610065, P. R. China; Universidad Rey Juan Carlos, SPAIN

## Abstract

This paper presents improvements to the conventional Topology Representing Network to build more appropriate topology relationships. Based on this improved Topology Representing Network, we propose a novel method for online dimensionality reduction that integrates the improved Topology Representing Network and Radial Basis Function Network. This method can find meaningful low-dimensional feature structures embedded in high-dimensional original data space, process nonlinear embedded manifolds, and map the new data online. Furthermore, this method can deal with large datasets for the benefit of improved Topology Representing Network. Experiments illustrate the effectiveness of the proposed method.

## Introduction

Techniques for dimensionality reduction have attracted much attention in many fields such as machine learning and data mining [1] [2] [3] [4] [5] [6] [7] [8] [9] [10]. Dimensionality reduction methods are used for mapping high-dimensional observations into desired low-dimensional space while preserving the features hidden in the original space. Over the past decades, a number of dimensionality reduction methods have been proposed. Principal Component Analysis (PCA) [11] [12] [13] [14] [15] [16] [17] [18] and Multidimensional Scaling (MDS) [19] [20] [21] have been the two most popular methods because of their relative simplicity and effectiveness. However, PCA is designed to operate when the manifold is embedded linearly or almost linearly in the subspace, and it cannot project previously “unseen” patterns. Classical MDS finds a low-dimensional embedding of patterns with distances in the target space that reflects dissimilarities in the original sample. Both PCA and MDS cannot disclose nonlinearly embedded manifolds because they operate on Euclidean distances. To overcome this limitation, many nonlinear methods have been proposed. Locally Linear Embedding (LLE) [22] maps high-dimensional original data feature space into a single global coordinate system of low dimensionality. Laplacian Eigenmap [23] uses spectral techniques to perform dimensionality reduction. ISOMAP [24] [25] employs classical MDS for geodesic distances in the original data feature space. L-ISOMAP [26] increases ISOMAP’s efficiency. It approximates a large global computation in ISOMAP by a much smaller set of calculations.

Because geodesic distances are especially suitable for computing distances among data points embedded in nonlinear manifolds, many methods to build graphs on the data have been proposed. The Topology Representing Network (TRN) [27] [28] [29] [30] is representative because of its effectiveness and simplicity. TRN, which combines the neural gas (NG) vector quantization method with the competitive Hebbian learning rule is used to quantize embedded manifolds and learn the topological relations of the input space without the necessity of prespecifying a topological graph. There are some dimensionality reduction methods based on TRN. Online data visualization using the neural gas network (OVI-NG) [31] is a distance preserving mapping of the codebook vectors (vector quantization) obtained by the NG algorithm. The codebook positions (codebook vectors’ projection in low-dimensional space) are adjusted in a continuous output space using an adaptation rule that minimizes a cost function that favors local distance preservation. OVI-NG is not able to disclose nonlinear embedded manifolds because of its use of Euclidean distances. The Geodesic Nonlinear Projection Neural Gas (GNLP-NG) algorithm [32] is an extension of OVI-NG that uses geodesic distances instead of Euclidean distances so that GNLP-NG performs well in the projection of nonlinear embedded manifolds. GNLP-NG and OVI-NG are not able to project new data. The method RBF-NDR [33], which includes the NG algorithm and RBFN, can process data online. Nonetheless, RBF-NDR sometimes has poor mapping quality and sometimes performs well due to minimizing STRESS [33] at each iteration without clear targets.

In this paper, we propose a new method for online and nonlinear dimensionality reduction called ITRN-RBF. We improve the conventional TRN so that it builds a more appropriate topology relationship. That is, the method we call the Improved TRN (ITRN) is more specifically suited to calculating geodesic distances. Furthermore, large amounts of data can be processed by ITRN’s vector quantization. We chose the MDS method as the mapping method. In contrast to classical MDS operating on Euclidean distances, our method operates on the geodesic distances of the topology graph reconstructed by ITRN. The mapping between the original high-dimensional space and low-dimensional feature structures embedded is then learned by supervised RBFN, whose target values are generated by the mapping methods. In particular, we give two implementations of RBFN. One is trained by the Widrow-Hoff learning algorithm. The other is an exact RBFN designed by precise mathematical calculation. Finally, the RBFN is used to reduce the dimensions of the original high-dimensional data. ITRN-RBF can process nonlinearly embedded manifolds, preserve the global structure of these manifolds, and project new data online.

## Methods

ITRN-RBF comprises two procedures: capturing the topology of the given dataset using ITRN and learning the mapping using RBF. The first procedure learns the topology of the input data embedded in the high-dimensional original data feature space and generates a graph using ITRN. ITRN connects the subgraphs together to ensure the connectivity of the resulting graph. The method for connecting the subgraphs is discussed in the section below. Using the output (codebook vectors with similarity relationships) from the first procedure, the second procedure calculates the pairwise graph distances as geodesic distances and constructs the mapping between the high-dimensional original space and low-dimensional target space. It then uses RBFN to learn this mapping. In particular, there are variety of ways to implement RBFN. We give two different implementations, which are described below. Finally, RBFN is just the dimensionality reduction tool, which has the desired capabilities of processing nonlinearly embedded manifolds and projecting new data online. In the following, ITRN-RBF is introduced and discussed in detail.

### ITRN

TRN is one of the vector quantization algorithms that are based on neural network models, which are capable of adaptively quantizing a given set of input data. Given a set of data *X* = {**x**_1_, **x**_2_, …, **x**_*N*_}, **x**_*j*_ ∈ *R* ^*D*^, TRN employs a finite set *V* = {**v**_1_, **v**_2_, …, **v**_*n*_}, **v**_*i*_ ∈ *R* ^*D*^ called codebook vectors (or reference vectors, neural units) to encode *X*. TRN learns the topological relation of *X* by distributing nodes among the data and connecting them using the competitive Hebbian rule. The purpose of TRN’s learning is to reconstruct a topology graph *G* = (*V*, *C*) for X, where *C* represents the adjacent matrix of *V*, whose values are constrained to 0 (unconnected) or 1 (connected). The conventional TRN algorithm operates as follows.

1. Set iteration step *t* = 0. Assign initial values to the codebook vectors **v**_*i*_(**v**_*i*_ ∈ *V*, *i* = 1, 2, …, *n*) and set all connection edges.
2. Randomly select input pattern **x** from *X*.
3. For each codebook vector **v**_*i*_, calculate rank *r*_*i*_ by determining the sequence (*i*_0_, *i*_1_, …, *i*_*n*−1_) by
  $$\|x-v_{i_{0}}\|<\|x-v_{i_{1}}\|<\cdots <\|x-v_{i_{n-1}}\|.$$ (1)
  That is, *r*_*i*_0__ = 0, *r*_*i*_1__ = 1, …, *r*_*i*_*n*−1__ = *n*−1.
4. Update all nodes **v**_*i*_ according to
  $$v_{i}^{new}=v_{i}^{old}+\epsilon \cdot e^{-r_{i}/\lambda \left(x-v_{i}^{old}\right)}.$$ (2)
5. Connect the two nodes closest to the randomly selected input pattern **x**. Set *c*_*i*_0_*i*_1__ = 1 and set this connection’s age to zero (*t*_*i*_0_*i*_1__ = 0).
6. Increase the age of all connections of **v**_*i*_0__ by setting *t*_*i*_0_*j*_ = *t*_*i*_0_*j*_ + 1 for all nodes **v**_*j*_ that are connected to node **v**_*i*_0__ (*c*_*i*_0_*j*_ = 1).
7. Remove the connections of node **v**_*i*_0__ that have exceeded their lifetime by setting *c*_*i*_0_*j*_ = 0 for all *j* with *c*_*i*_0_*j*_ = 1 and *t*_*i*_0_*j*_ > *T*.
8. Increase the iteration step *t* = *t* + 1. If the maximum number of iterations has not yet been reached (*t* < *t*_*max*_), continue with step 2.

There are many parameters in this algorithm. The codebook vectors’ number *n* and maximum number of iterations *t*_*max*_ are both set by the user. The parameter *λ*, step size *ϵ* and lifetime *T* depend on the number of iterations. The time dependent parameters are set according to the form

$$g\left(t\right)=g_{i}{\left(\frac{g_{f}}{g_{i}}\right)}^{\frac{t}{t_{max}}}.$$ (3)

Here, *g*_*i*_ is the initial value of the variable, *g*_*f*_ is the final value, *t* denotes the iteration step and *t*_*max*_ represents the maximum number of iterations. Suggestions as to how to tune these parameters have been proposed by Martinetz and Schulten [27].

In fact, to obtain a denser graph that is better for calculating geodesic distances, we implement some improvements. For the randomly selected input patterns at each iteration, the method ITRN creates a connection between the 1_*st*_ and (*k* + 1)_*th*_ nearest nodes (1 ⩽ *k* ⩽ *kn*, typically *kn* ∈ {2, 3, 4}) instead of only connecting the first and second closest codebook vectors. In addition, we also connect the subgraphs to avoid the existence of infeasible nodes. Specific details about ITRN are presented in the statements below. Steps 1–5 are the same as steps 1–5 in the conventional TRN, hence we only list the steps that follow.

6. If the following condition is satisfied
  $$\|v_{i_{s}}-v_{i_{k}}\left\|=\min(\right\|v_{i_{0}}-v_{i_{k}}\left\|,\right\|v_{i_{1}}-v_{i_{k}}\left\|,\ldots ,\right\|v_{i_{k-1}}-v_{i_{k}}\left\|\right)$$ (4)
  for *k* = 1, 2, …, *kn*, then create a connection between nodes **v**_*i*_*s*__ *and* **v**_*i*_*k*__ by setting *c*_*i*_*s*_*i*_*k*__ = 1 and *t*_*i*_*s*_*i*_*k*__ = 0.
7. Increase the age of all connections of **v**_*l*_(*l* = *i*_0_, *i*_1_, …, *i*_*kn*−1_) by setting *t*_*lj*_ = *t*_*lj*_ + 1 for all nodes **v**_*j*_ that are connected to node **v**_*i*_0__ (*c*_*i*_0_*j*_ = 1).
8. Remove the connections of node **v**_*l*_(*l* = *i*_0_, *i*_1_, …, *i*_*kn*−1_) that have exceeded their lifetime by setting *c*_*lj*_ = 0 for all *j* for which *c*_*lj*_ = 1 and *t*_*lj*_ > *T*.
9. Increase the iteration step:*t* = *t* + 1. If the maximum number of iterations has not yet been reached (*t* < *t*_*max*_), continue with step 2.
10. If the resulting graph *G* = (*V*, *C*) is unconnected, it is necessary to connect the subgraphs. Assume that *G* = {*G*_1_, *G*_2_, …, *G*_*c*_}, where *G*_*i*_ is the subgraph that is not connected to the others. Calculate *E* = **e**_*ij*_, where *e*_*ij*_ is the shortest edge obtained by connecting the closest nodes in *G*_*i*_ and *G*_*j*_. Finally, choose a suitable **e**_*ij*_ to add to *C* and obtain the connected graph *G* ^*E*^ = (*V*, *C* ^*E*^).

**Fig 1:**
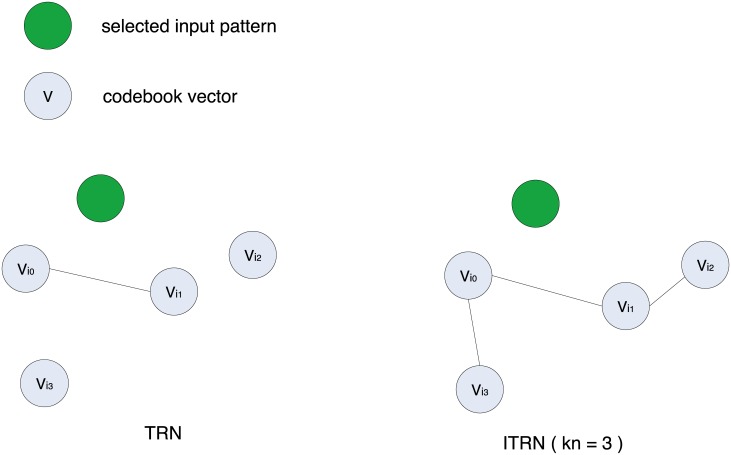
Different strategies to establish connections.

**Fig 2:**
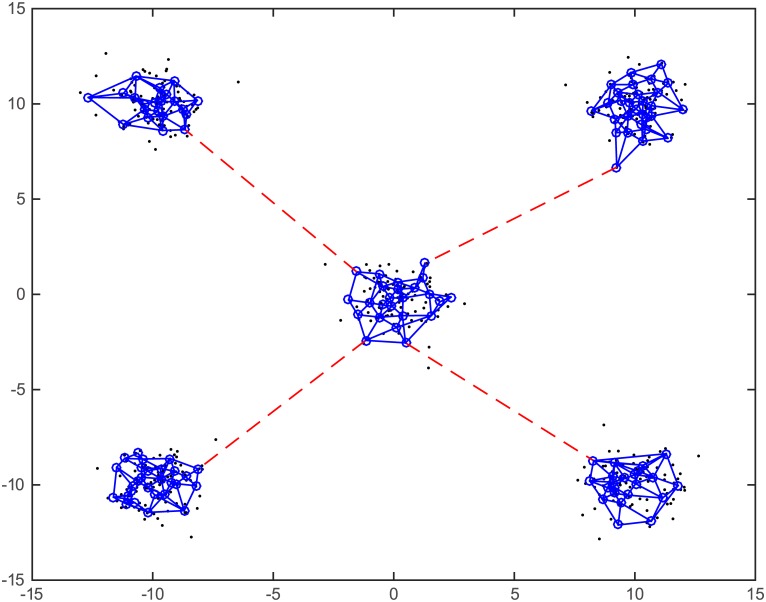
Connecting the subgraphs in ITRN step 10. The dataset is formed of randomly generated nodes comprising five non-overlapping clusters (S1 Dataset). Black dots indicate the training patterns (500 nodes), and blue circles indicate the codebook vectors (100 vectors). In addition, the blue solid lines are established by ITRN steps 1–9 and the dotted lines are established by ITRN step 10.

**Fig 3:**
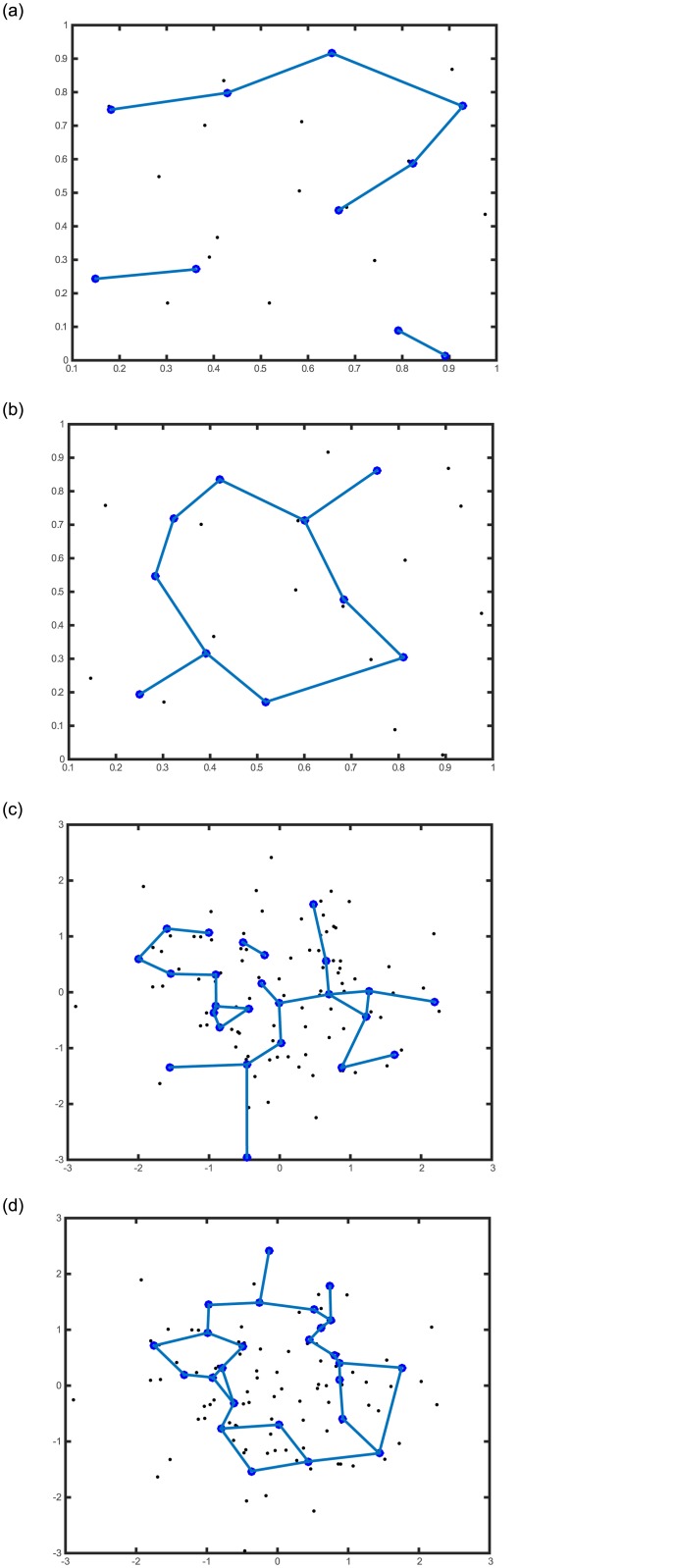
Comparison of TRN and ITRN. Black dots indicate the training patterns, and blue circles indicate codebook vectors. In the first experiment, 20 randomly generated training patterns (S1 Dataset) and 10 codebooks were selected, and (a) and (b) show the results generated by TRN and ITRN, respectively. In the second experiment, 100 randomly generated training patterns (S1 Dataset) and 25 codebooks were selected, and (c) and (d) show the results generated by TRN and ITRN, respectively.

**Fig 4:**
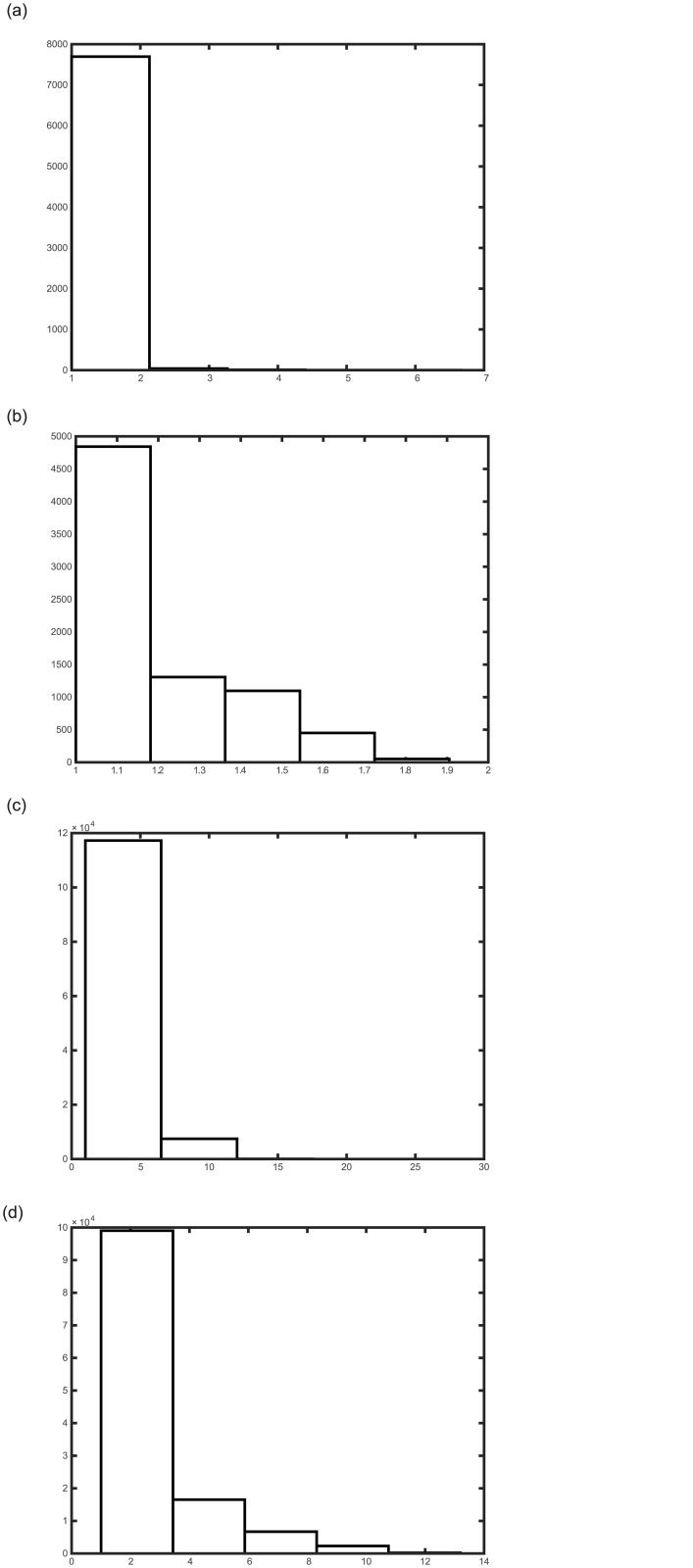
Comparison of distance ratio. (a) and (b) show the ratios for TRN and ITRN, respectively, calculated with an artificial point set, and (c) and (d) show the ratio s for TRN and ITRN, respectively, calculated with a Swiss roll dataset.

**Fig 5:**
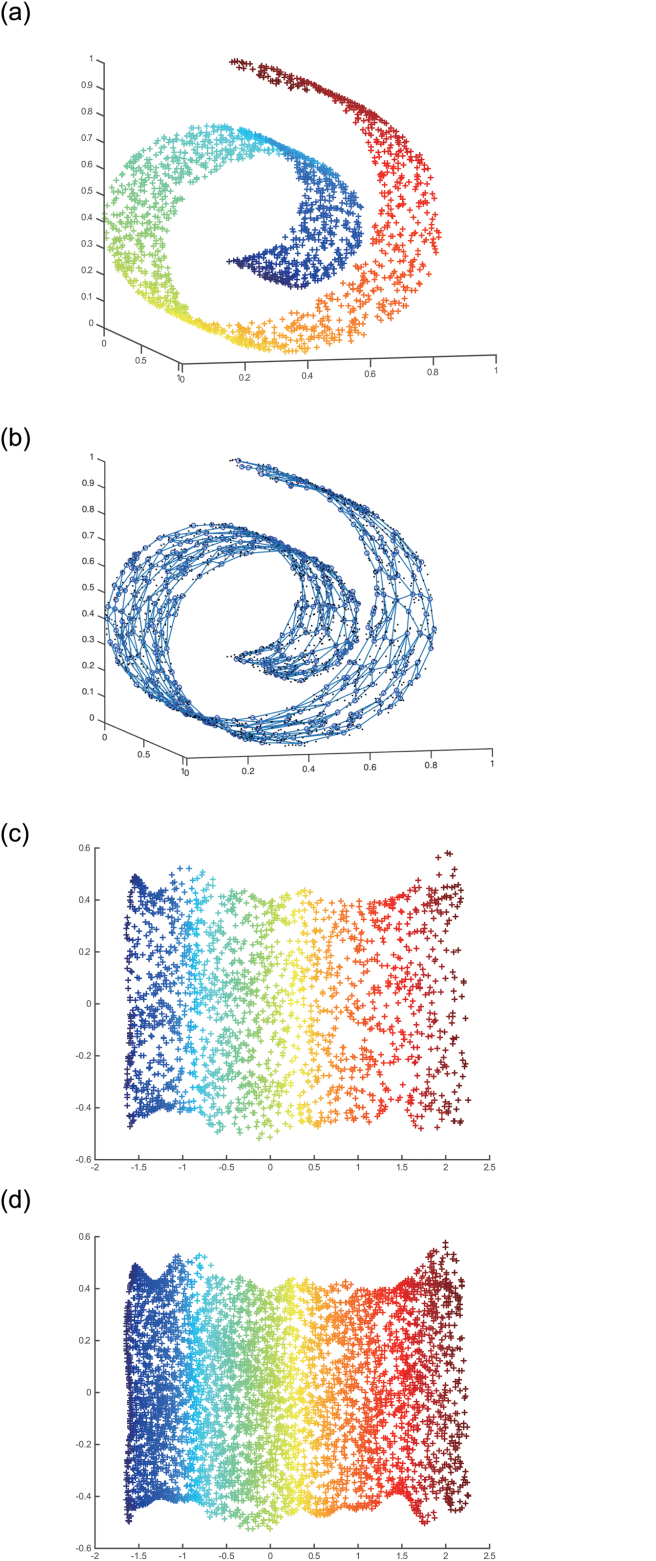
ITRN-ERBF results for Swiss roll. (a) shows Swiss roll dataset, (b) shows learing result by ITRN, (c) shows mapping of the training patterns, (d) shows mapping of the new dataset.

Compared with conventional TRN, we note that:

- ITRN modifies the TRN strategy to establish the connections in steps 6–8 (see Fig 1) and connect subgraphs in step 10 (see Fig 2).
- Conventional TRN causes deviation because it ignores some topological relations of the codebook vectors. However, ITRN connects multiple points so that more topological relations can be established. In addition, a relation caused by miscalculation will be removed when its lifetime exceeds the limit. An experiment shows the different construction, as shown in Fig 3.
- The distance ratio defined as follows:
  $$ratio=\frac{GD_{ij}}{ED_{ij}}$$ (5)
  can be used to quantitatively evaluate the connection quality. Where *GD*_*ij*_ denotes the geodesic distance and *ED*_*ij*_ denotes the Euclidean distance between codebook vectors **v**_*i*_ and **v**_*j*_. The bar chart shown in Fig 4 displays the statistical results (the x-axis is distance ratio interval and y-axis is the node number). Fig 4a and 4b are based on the dataset that is shown in Fig 2. Fig 4c and 4d use the Swiss roll dataset (shown in Fig 5a). The ITRN’s bar chart has a larger gradient and much more restricted *ratio* range, both of which are desirable.

### RBFN

In this section, we propose two methods to train or design an RBFN. The first approach, called the training RBFN (TRBF), is a D-h-d network that includes an input layer with D units (equal to the codebook vectors’ dimensionality), hidden layers with h units (set by users), and an output layer with d units (equal to the dimensionality of the output space). The second approach, named exact RBFN (ERBF), is a D-n-d network with the same parameters as the training RBFN. Especially the number of hidden layer units n is equal to the number of codebook vectors. All of them have the same codebook vector input s obtained by ITRN and the same training targets given by MDS. What is more important, MDS is based on geodesic distances that are calculated from the graph *G* ^*E*^ = (*V*, *C* ^*E*^) and the training targets defined as *T* = {**t**_1_, **t**_2_, …, **t**_*n*_}, **t**_*i*_ ∈ *R* ^*d*^ are certain, so we can obtain a stable RBFN. For more details, the interested reader can refer to [34] [35] [36] [37] [38] [39].

#### TRBF

In terms of TRBF, we chose a Gaussian function as the activation function, defined as follows:

$$\phi_{i}\left(x_{j}\right)=e^{-\frac{1}{2}\frac{\|x_{j}-c_{i}{\|}^{2}}{\sigma_{i}^{2}}}=e^{-\frac{1}{2}\sum_{l=1}^{D}\frac{{\left(x_{lj}-c_{li}\right)}^{2}}{\sigma_{li}^{2}}}.$$ (6)

The hidden layer output is defined as

$$H=\left\{h_{1},h_{1},\ldots ,h_{h}\right\},h_{ij}=\phi_{i}\left(v_{j}\right),i\in \left[1,h\right],j\in \left[1,n\right].$$ (7)

In addition, the loss function is given by

$$E_{j}=\frac{1}{2}e_{j}^{2}=\frac{1}{2}{\left\|t_{j}-y_{j}\right\|}^{2}=\frac{1}{2}\sum_{k=1}^{d}{\left(t_{kj}-y_{kj}\right)}^{2}.$$ (8)

The TRBF network provides four types of adjustable parameters: center *c*_*li*_, widths *σ*_*li*_, weights *w*_*ik*_ and bias **b**_*k*_. Based on the Widrow-Hoff learning algorithm, the calculation equations of each parameter are given by:

$$c_{li}=c_{li}+\eta_{c}\sum_{k=1}^{d}\left(t_{kj}-y_{kj}\right)\frac{w_{ik}}{\sigma_{li}^{2}}\phi_{i}\left(x_{j}\right)\left(x_{lj}-c_{li}\right),l\in \left[1,D\right],i\in \left[1,h\right],$$ (9)

$$\sigma_{li}=\sigma_{li}+\eta_{\sigma }\sum_{k=1}^{d}\left(t_{kj}-y_{kj}\right)\frac{w_{ik}}{\sigma_{li}^{3}}\phi_{i}\left(x_{j}\right){\left(x_{lj}-c_{li}\right)}^{2},l\in \left[1,D\right],i\in \left[1,h\right],$$ (10)

$$w_{ik}=w_{ik}+\eta_{w}\left(t_{kj}-y_{kj}\right)e^{-\frac{1}{2}\sum_{l=1}^{D}\frac{{\left(x_{lj}-c_{li}\right)}^{2}}{\sigma_{li}^{2}}},i\in \left[1,h\right],k\in \left[1,d\right]$$ (11)

$$b_{k}=b_{k}+\eta_{b}\left(t_{kj}-y_{kj}\right),k\in \left[1,d\right],$$ (12)

where *η*_*c*_, *η*_*σ*_, *η*_*w*_, and *η*_*b*_ which are individual step sizes for *c*_*li*_, *σ*_*li*_, *w*_*ik*_, and **b**_*k*_, respectively, can be defined by users.

#### ERBF

ERBF’s weight *W* and output layer bias *B* are obtained by mathematical calculation, so the RBFN can ensure zero error, in theory. The linear equations are given as follows:

$$\left\{W,B\right\}\cdot {\left\{H,\text{ones}\right\}}^{T}=T.$$ (13)

The input layer bias *b*_*in*_ is set as $\sqrt[{2}]{-log0.5}/spread$ so there is only one parameter that needs to be set by users. How to set the *spread* is described in the results section.

### ITRN-RBF method

The detailed algorithm process is as follows:

1. Construct graph *G* ^*E*^ = (*V*, *C* ^*E*^) using ITRN. In reality, the graph is connected.
2. Calculate the geodesic distances on *G* ^*E*^.
3. Construct the mapping between the high-dimensional original space and low-dimensional target space by using MDS operating on the geodesic distances of the topology graph. For every **v**_*j*_, we get output **t**_*j*_ as an expectation.
4. Train or design an RBFN with explicit inputs *V* and outputs *T*. In this step, any appropriate RBFNs such as ERBF or TRBF could be applied.
5. Use the RBFN to map the dataset.

## Results

In this section, ITRN-RBF is used for visualization and feature extraction, and is also compared with others including methods based on TRN and classical dimensionality reduction methods such as ISOMAP, L-ISOMAP and PCA. We also present the computational complexity analysis of the method and a table with running times.

There are many parameters for experimental data. The common parameters of TRN, OVI-NG and GNLP-NG were set as follows: *t*_*max*_ = 20*n*, *ϵ*_*i*_ = 0.1, *ϵ*_*f*_ = 0.05, *λ*_*i*_ = 0.05*n*, *λ*_*f*_ = 0.01, *T*_*i*_ = 0.05*n*, and *T*_*f*_ = *n*. The auxiliary parameters of the OVI-NG and GNLP-NG were set as *α*_*i*_ = 0.3, *α*_*f*_ = 0.001, *σ*_*i*_ = 0.7*n*, and *σ*_*f*_ = 5. The extra parameter for ITRN *kn* was set to two (for the Swiss roll) or three (for the artificial faces, handwritten digit “2” and UMist faces datasets). The parameters of RBFN in the Swiss roll experiment were set as follows: *η*_*c*_ = 0.03, *η*_*σ*_ = 0.03, *η*_*w*_ = 0.2. For the image processing experiments, they were changed to *η*_*c*_ = 0.002, *η*_*σ*_ = 0.002, and *η*_*w*_ = 0.05. The ERBF’s parameter *spread* can be obtained as follows:

$$spread=max(d_{ij}),$$ (14)

where *d*_*ij*_ denotes the Euclidean distances between the codebook vectors. The number of neighbors used in the compuations for ISOMAP and L-ISOMAP is set to 12. The number of landmarks used in L-ISOMAP is set to 0.1*n*.

### Comparison with the methods based on TRN

We chose two standard metrics for mapping quality. They are widely used for analysing dimensionality reduction methods based on TRN.

- Distance preservation: This value evaluates the distance difference between nodes in input space and nodes in output space. We chose the classical MDS [19] [20] and Sammon stress functions [40] to quantify this value. Their expressions are as follows:
  $$E_{MDS}=\sum_{i<j}^{n}{\left(d_{ij}-{\hat{d}}_{ij}\right)}^{2},$$ (15)
  $$E_{SM}=\frac{1}{\sum_{i<j}^{n}d_{ij}}\sum_{i<j}^{n}\frac{{\left(d_{ij}-{\hat{d}}_{ij}\right)}^{2}}{d_{ij}},$$ (16)
  where *d*_*ij*_ is the distance between nodes in the original space and ${\hat{d}}_{ij}$ is the distance between nodes in output space. Moreover, when the mapping method uses geodesic distances, the expression is calculated using geodesic distances. Otherwise, the method uses Euclidean distances.
- Neighborhood preservation: This value evaluates the degree to which adjacent patterns in input space are close in output space. The measures of trustworthiness *M*_1_(*k*) and continuity *M*_2_(*k*) [41] [42] are suitable. Their expressions are given below:
  $$M_{1}\left(k\right)=1-\frac{2}{nk(2n-3k-1)}\sum_{i=1}^{n}\sum_{v_{j}\in U_{k}\left(v_{i}\right)}\left(r_{ij}-k\right),$$ (17)
  $$M_{2}\left(k\right)=1-\frac{2}{nk(2n-3k-1)}\sum_{i=1}^{n}\sum_{v_{j}\in V_{k}\left(v_{i}\right)}\left({\hat{r}}_{ij}-k\right),$$ (18)
  where *U*_*k*_(**v**_*i*_) is the set of nodes that are in the *k*-size neighborhood of the codebook vector *i* in the output space but not in the original space. In contrast, *V*_*k*_(**v**_*i*_) denotes the set of nodes that belong to the *k*-size neighborhood of codebook vector *i* in the original space rather than in output space. Rank *r*_*ij*_ refers to rank in the original space, but ${\hat{r}}_{ij}$ denotes the order in output space. In fact, trustworthiness and continuity are functions of the number of neighbors *k*.

Three methods, OVI-NG, GNLP-NG, and RBF-NDR, were selected for comparison. In particular, OVI-NG and GNLP-NG can only map the codebook vectors. Hence, to keep the comparison fair, we used the RBFN obtained by RBF-NDR and ITRN-RBF to map the codebook for comparison. All methods’ line charts with respect to trustworthiness and continuity are given after each experiment, except for OVI-NG, because the method cannot process nonlinear embedded manifolds. (We only show the results separately in the Swiss roll experiment.) Table 1 presents the stress functions for the different methods.

**Table 1:** Stress functions for different methods.

Methods	Swiss roll		AF		“2”	
	*E*_*MDS*_	*E*_*SM*_	*E*_*MDS*_	*E*_*SM*_	*E*_*MDS*_	*E*_*SM*_
ITRN-ERBF	2.5204E+03	0.0094	6.2399E+03	0.0125	5.4540E+07	0.9379
ITRN-TRBF	3.6855E+03	0.0153	1.3094E+04	0.0295	4.8321E+07	0.9851
RBF-NDR	2.6040E+03	0.0114	3.1725E+04	0.0812	4.4090E+06	0.0892
GNLP-NG	3.4730E+03	0.0116	2.6486E+04	0.0507	4.6615E+06	0.1064

#### Swiss roll

The Swiss roll (S2 Dataset) corresponds to a two-dimensional pattern distributed uniformly on a plane and embedded nonlinearly in 3D (Fig 5a). We used ITRN to learn this manifold and ensure the connectivity of the resulting graph. The graph given in Fig 5b shows the reconstructed manifold embedded in the high-dimensional original data feature space by ITRN. We then trained an RBFN to reduce the dimensionality. The projection estimated by the ERBF module is given in Fig 5c and 5d. Fig 5c shows the mapping of the training pattern (2000 nodes), and Fig 5d shows the mapping of the new dataset (5000 nodes) that was taken from the Swiss roll by random sampling. We observe that TRN-RBF is able to recover the intrinsic two-dimensionality of the Swiss roll and process a new dataset.

**Fig 6:**
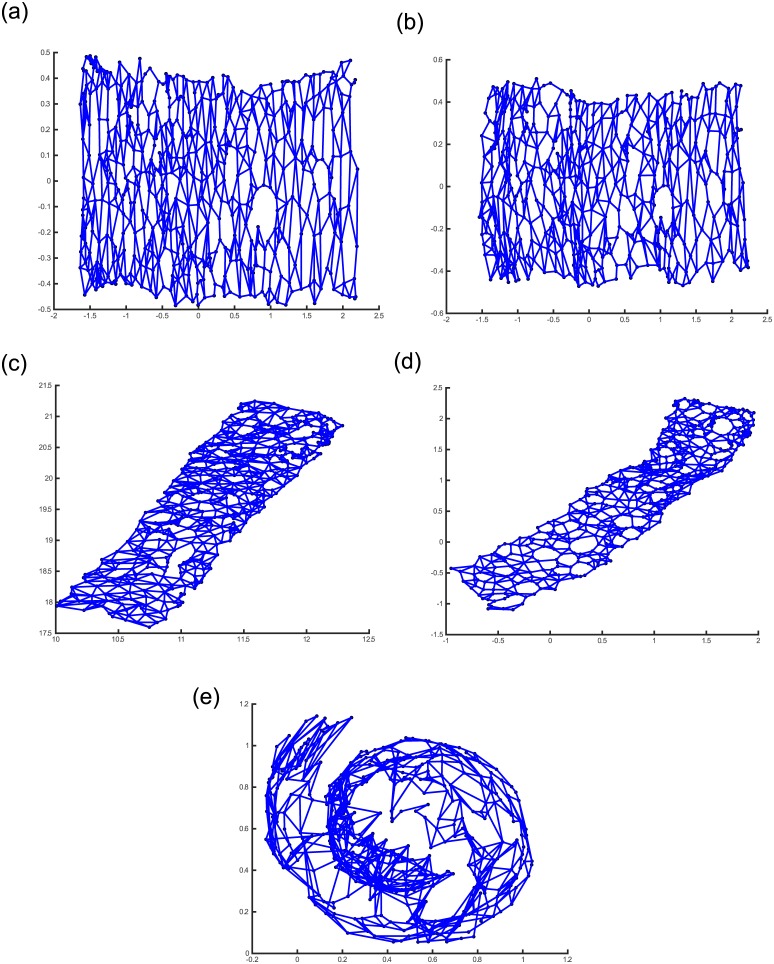
Different mappings of the Swiss roll’s codebook vectors for different methods. (a) ITRN-ERBF, (b) ITRN-TRBF, (c) RBF-NDR, (d) GNLP-NG, and (e) OVI-NG.

**Fig 7:**
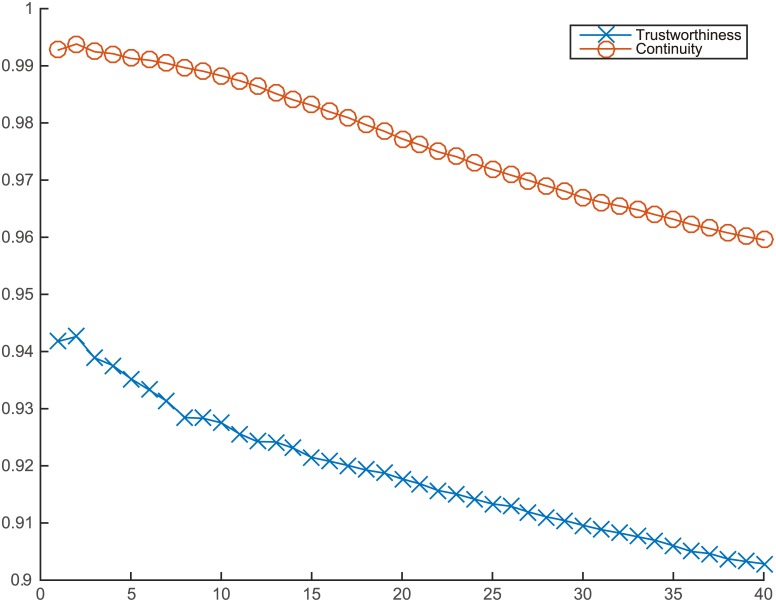
OVI-NG mapping quality for Swiss roll.

The different mappings of the Swiss roll’s codebook vectors are presented in Fig 6. All methods disclose the embedded manifolds of the Swiss roll except OVI-NG. The neighborhood preservation achieved by OVI-NG is presented in Fig 7. This method shows such a poor performance, only ITRN-RBF, RBF-NDR, and GNLP-NG are discussed in the following. Moreover, for RBF-NDR and GNLP-NG, the purpose of iterative adjustment is to minimize the stress function, hence they have similar mapping structures.

**Fig 8:**
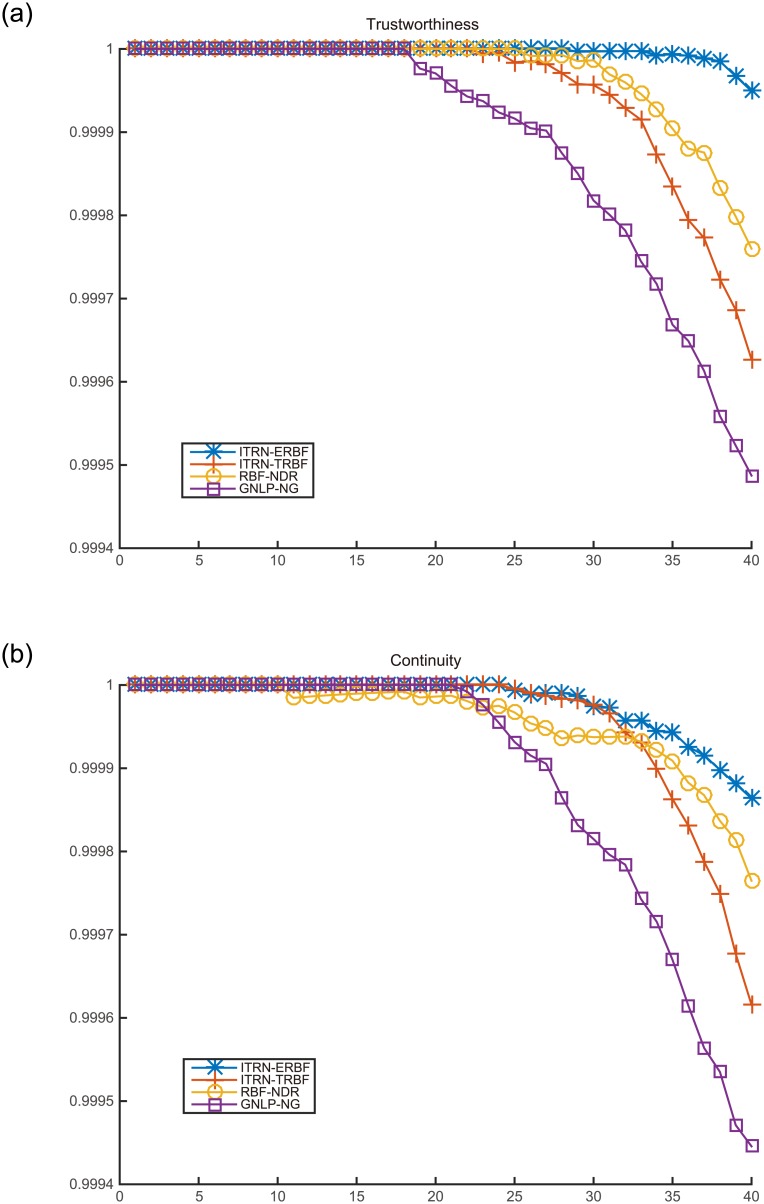
Mapping quality for the Swiss roll.

Analyzing each of the measures shown in Fig 8 and Table 1, it is clear that ITRN-ERBF retains two distinct advantages with respect to distance and neighborhood preservation. Closest to ITRN-ERBF in performance is RBF-NDR. Methods GNLP-NG and ITRN-TRBF perform almost as well.

#### Artificial and real-world images

The artificial images (S3 Dataset) are from the domain of visual perception. The dataset contains 698 artificially generated images of faces (image size: 64 × 64, 688 images for training and 10 for testing, referred to as AFs) under different poses and different illumination conditions.

The real-world images (S4 Dataset) come from the Mixed National Institute of Standards and Technology (MNIST) database. We chose the handwritten digit “2” (image size: 28 × 28, 1000 images for training and 10 for testing, referred to as “2”) for this experiment because of its varied forms.

In particular, for the different datasets, there are two treatments: AF are preprocessed by PCA. The principal components that contribute less than 0.1% to the explained variance are discarded, hence dimensionality reduction methods are used for mapping the primed dataset. However, for “2,” we chose the original dataset as the training patterns.

**Fig 9:**
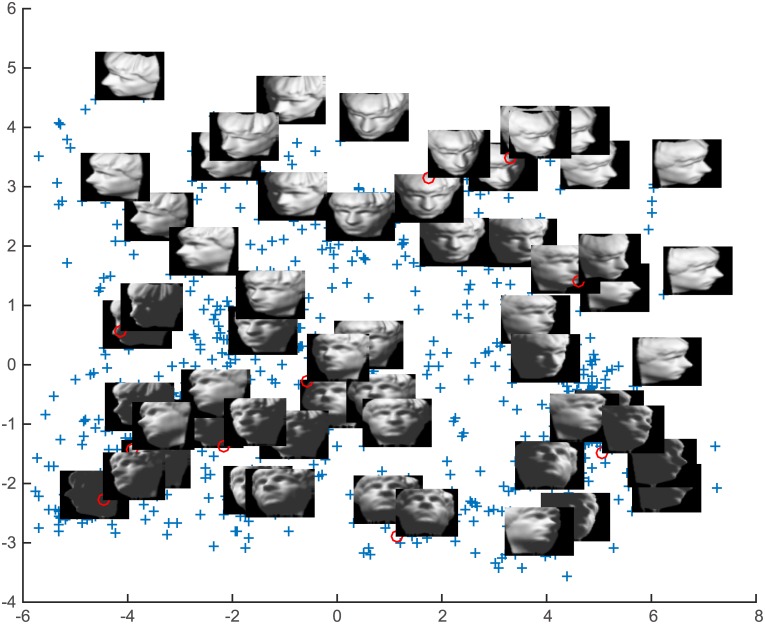
AF results.

**Fig 10:**
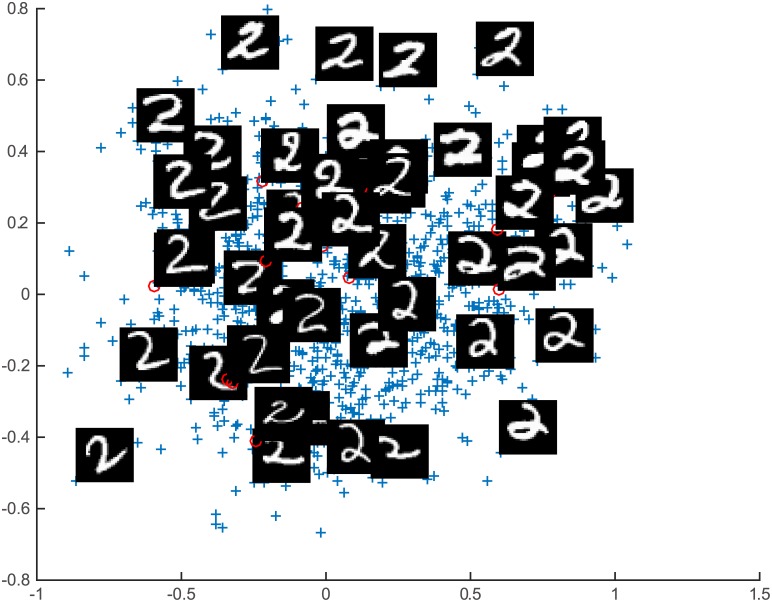
Handwritten digit “2” results.

**Fig 11:**
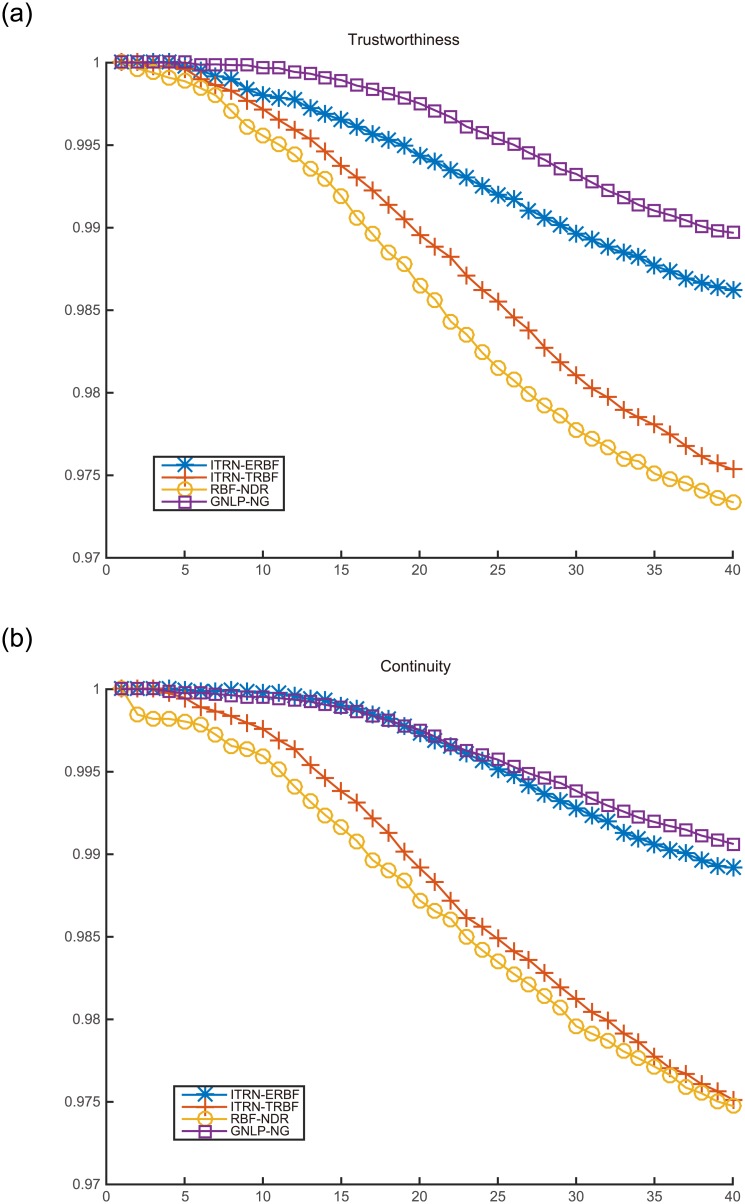
Mapping quality for AFs.

**Fig 12:**
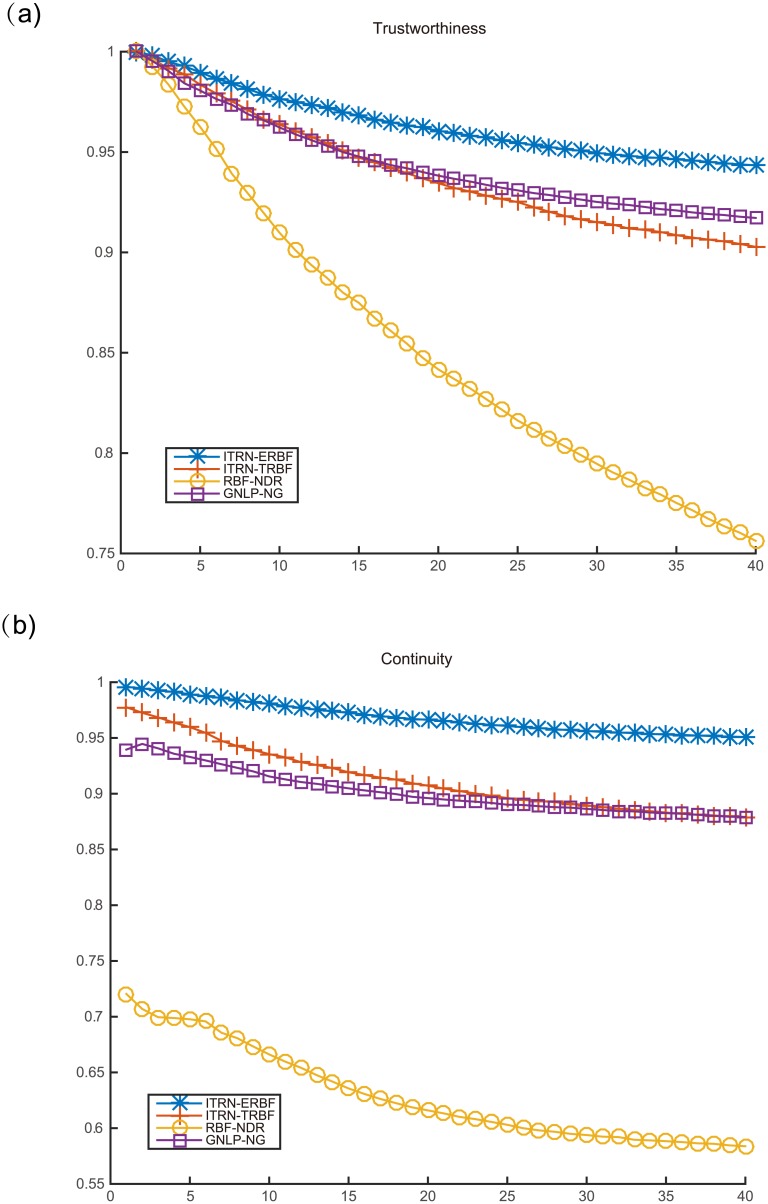
Mapping quality for handwritten digit “2”.

ITRN-ERBF and other methods were used for the task of visual perception. The resulting two-dimensional projection of training patterns obtained by ITRN-ERBF is given in Figs 9 and 10. A comparison of the mapping quality is presented in Figs 11 and 12 as well as Table 1. Blue plusses represent the two-dimensional projections of training patterns and red circles represent testing patterns’ position. For easy inspection, only part of the training patterns’ corresponding images were plotted. The major articulation features of the AF, left-right (x-axis) and up-bottom (y-axis), are captured from the input space. For the “2” dataset, the bottom loop (x-axis) and lean (y-axis) are captured from input space.

In terms of mapping quality, ITRN-ERBF has a high adaptability and performance. In contrast, ITRN-TRBF, GNLP-NG, and RBF-NDR perform less well. In very rare cases, GNLP-NG shows the best distance preservation feature because the goal of GNLP-NG is to minimize the stress function.

#### Comparison with RBF-NDR

Most dimensionality reduction methods can process new datasets because of RBFN. However, an imprecise RBFN could lead to imprecise projections. Hence, ITRN-ERBF, ITRN-TRBF, and RBF-NDR were selected to determine whether they were able to generate definitive results. All of them use RBFN to project the dataset.

**Fig 13:**
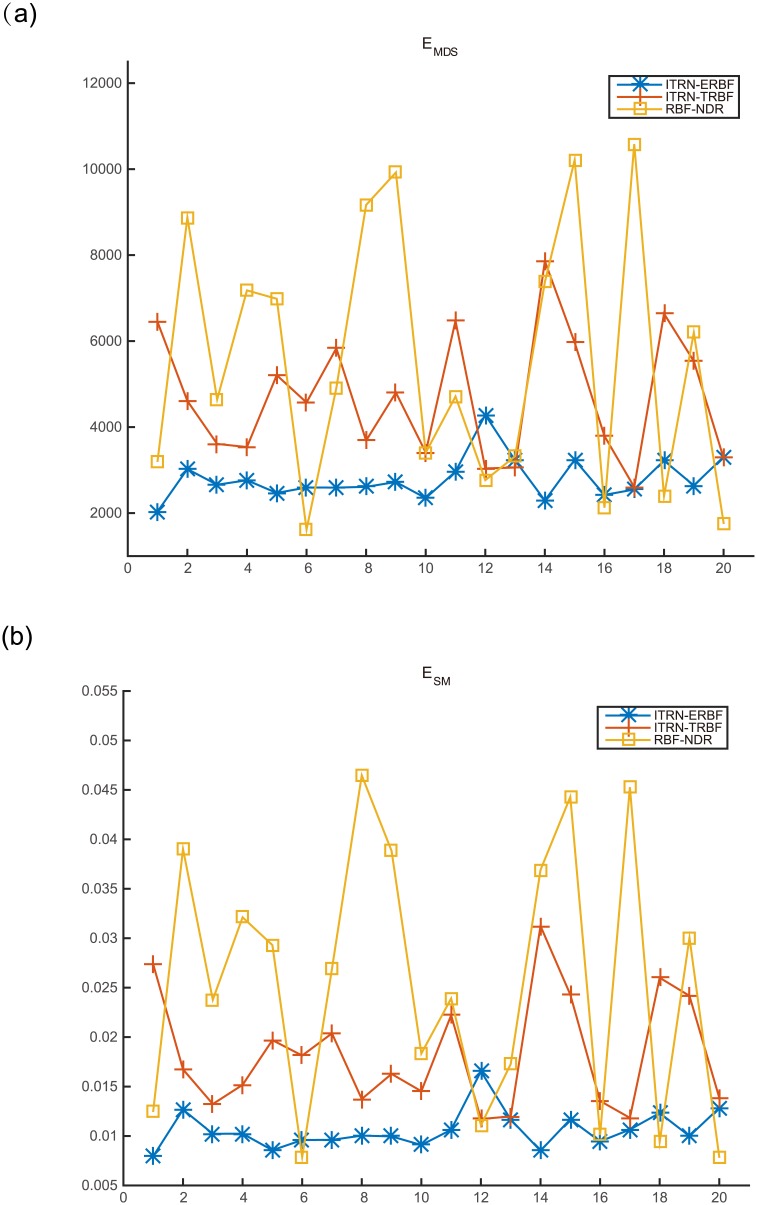
Comparison with RBF-NDR.

All methods ran 20 times on a uniform Swiss roll dataset. At each iteration, the manifold learning procedure was executed afresh and the RBFN was also designed or trained again. The results are shown in Fig 13. Here, the x-axis denotes the iterations and the y-axis represents the value of *E*_*MDS*_ or *E*_*SM*_. We observe that ITRN-ERBF has the smoothest line, indicating that ITRN-ERBF has the most definitive results. In contrast, ITRN-TRBF and RBF-NDR have obvious fluctuations because of their trained RBFN, which could not minimize the stress function or loss function.

### Comparison against the classical methods

In this section, ITRN-RBF was compared with classical dimensionality reduction methods including ISOMAP, L-ISOMAP, PCA. Three quality metrics [43], namely, stress function, the correlation coefficient, smooth neighborhood preservation were used for analysis. We detail the three quality metrics in the following.

- Stress function. You can refer to Eq 16.
- Correlation coefficient. This value measures how distances in the original space are correlated to those in the visual space. The expression are as follows:
  $$E_{CC}=1-\frac{<D⊙\hat{D}>-<D><\hat{D}>}{\sigma_{D}\sigma_{\hat{D}}},$$ (19)
  where *D* and $\hat{D}$ are the upper triangular distance metrics before and after projection, ⊙ is the element-by-element product, <> is the average operator and *σ* is the standard deviation of the vector’s elements. The smaller the value of *E*_*CC*_, the better the performance of the visualization is.
- Smooth neighborhood preservation. This is also a neighborhood preservation metric, but it’s based on distance instead of rank order compared with trustworthiness and continuity. The local misplacing metrics are defined as follows:
  $$W^{T}\left(v_{i}\right)=\{\begin{array}{cc} \frac{1}{|N^{T}\left(v_{i}\right)|}\sum_{v_{j}\in N^{T}\left(v_{i}\right)}w\left({\hat{r}}_{i},{\hat{d}}_{ij}\right) & N^{T}\left(v_{i}\right)\neq \phi ,\\ 0 & others, \end{array}$$ (20)
  $$W^{FN}\left(v_{i}\right)=\{\begin{array}{cc} \frac{1}{|N^{FN}\left(v_{i}\right)|}\sum_{v_{j}\in N^{FN}\left(v_{i}\right)}w\left(r_{i},d_{ij}\right) & N^{FN}\left(v_{i}\right)\neq \phi ,\\ 0 & others, \end{array}$$ (21)
  where *N* ^*T*^(**v**_*i*_) is the set of nodes in the *k*-nearest neighborhood (we set *k* = 12 for this analysis) of an node *i* that are not mapped among the *k*-nearest neighbors of *i* in the output space and *N* ^*FN*^(**v**_*i*_) is the set of nodes that are not among the *k*-nearest neighbors of *i* but are mapped among the *k*-nearest neighbors of *i* in the output space, |*W*| is the number of elements in the set and *w*(*r*, *t*) is given below:
  $$w\left(r,t\right)=\{\begin{array}{cc} \frac{28}{5}{\left(\frac{t-r}{r}\right)}^{5}-14{\left(\frac{t-r}{r}\right)}^{4}+\frac{46}{5}{\left(\frac{t-r}{r}\right)}^{3}+\frac{1}{5}{\left(\frac{t-r}{r}\right)}^{2} & r⩽t⩽2r,\\ 1 & others. \end{array}$$ (22)
  Smooth Neighborhood preservation can be obtained by simply computing:
  $$E_{NP}=\frac{1}{2|S|}\sum_{v_{i}\in S}\left(W^{T}\left(v_{i}\right)+W^{FN}\left(v_{i}\right)\right),$$ (23)
  where *S* is the set of nodes under analysis. The smaller the value of *E*_*NP*_ means the better neighborhood preservation.

**Fig 14:**
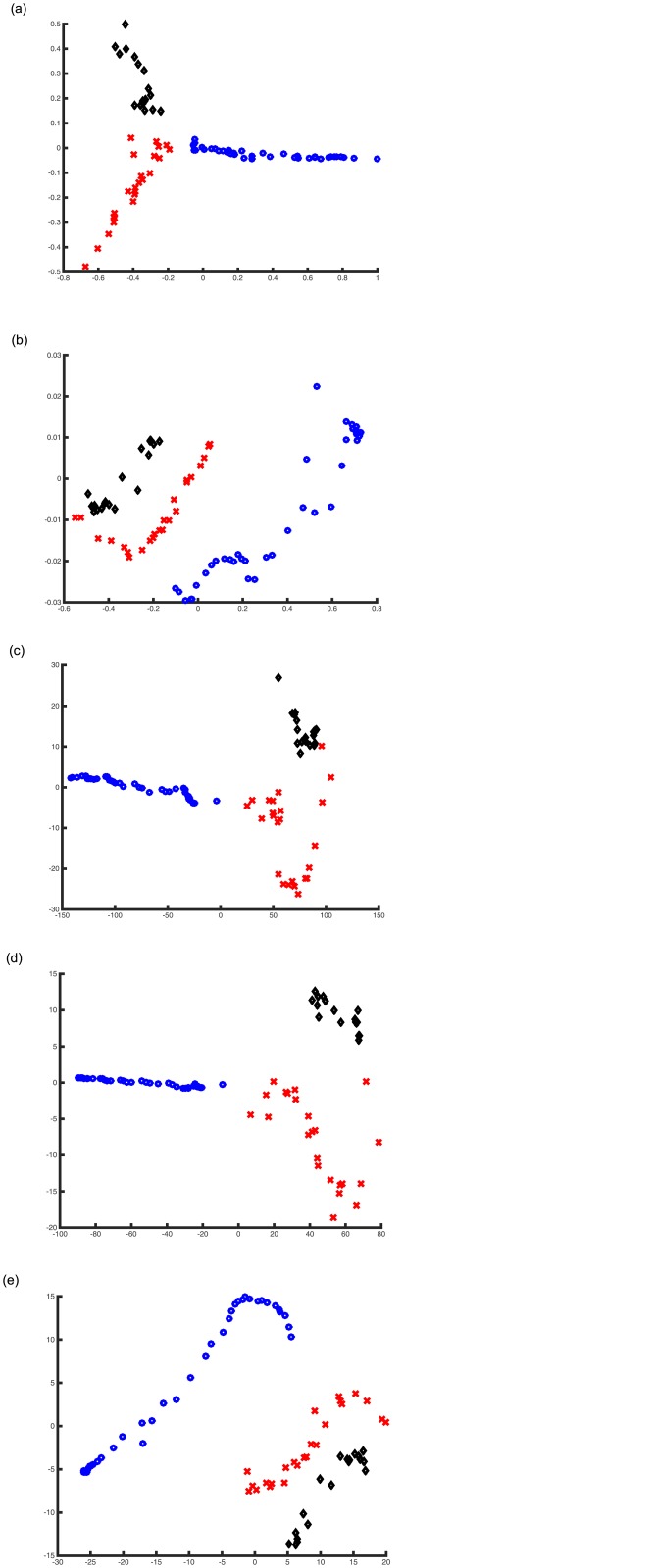
Visualizations of the UMist faces dataset. Different people’s faces denote different marks (black rhomb, red cross, blue circle). (a) ITRN-ERBF, (b) ITRN-TRBF, (c) ISOMAP, (d) L-ISOMAP and (e) PCA.

We add a dataset, three people’s face images (S5 Dataset) in UMist Faces database (575 total images, 112 × 92 size, manually cropped by Daniel Graham [44]), for showing feature extraction (Fig 14). Table 2 presents the quality metrics’ value for the different methods. We observe that PCA has poor performance because of nonlinear datasets. ISOMAP is better than L-ISOMAP because L-ISOMAP approximates a large global computation. ITRN-ERBF is better than ITRN-TRBF because ITRN-TRBF is trained and it has less center nodes in network. ITRN-RBF, ISOMAP and L-ISOMAP have similar results. In some cases, ITRN-RBF performs better than ISOMAP and L-ISOMAP. That illustrates the effectiveness of ITRN-RBF.

**Table 2:** Values of quality metrics for ITRN-RBF and classical dimensionality reduction methods.

Swiss roll					
Quality Metrics	ITRN-ERBF	ITRN-TRBF	ISOMAP	L-ISOMAP	PCA
*E*_*CC*_	6.7798E-04	0.0067	3.0124E-04	5.3887E-04	0.1536
*E*_*NP*_	0.0493	0.1082	0.1330	0.1537	0.7373
*E*_*SM*_	0.0094	0.0153	0.0012	0.0923	0.4921
AF					
Quality Metrics	ITRN-ERBF	ITRN-TRBF	ISOMAP	L-ISOMAP	PCA
*E*_*CC*_	0.0313	0.2569	0.1760	0.1326	0.1111
*E*_*NP*_	0.3271	0.4159	0.4752	0.4199	0.5887
*E*_*SM*_	0.0152	0.0295	0.0857	0.1608	0.1700
“2”					
Quality Metrics	ITRN-ERBF	ITRN-TRBF	ISOMAP	L-ISOMAP	PCA
*E*_*CC*_	0.2069	0.3200	0.2316	0.2641	0.4014
*E*_*NP*_	0.5184	0.5504	0.5657	0.5837	0.5806
*E*_*SM*_	0.9379	0.9851	0.1219	0.2529	0.3814
UMist face					
Quality Metrics	ITRN-ERBF	ITRN-TRBF	ISOMAP	L-ISOMAP	PCA
*E*_*CC*_	0.0057	0.0429	0.0080	0.0051	0.0583
*E*_*NP*_	0.0252	0.1813	0.1788	0.1129	0.1729
*E*_*SM*_	0.9877	0.9840	0.0138	0.1177	0.1305

### Computational complexity analysis

Assume that input space’s nodes number is *N*, codebook vectors number is *n*, TRN’s epochs is *k*_1_ and TRBF’s epochs is *k*_2_. The most time consuming part of TRN corresponds to sorting the distances for rank *r*_*i*_ which goes with *O*(*Nlog*_2_ *N*). Our improvement of TRN increases time cost because of building connecting graph. The extra time cost is *O*(*n* ^2^). However, in most applications, this time cost can be neglectable because of the small value of *n*. The MDS has complexity *O*(*n* ^3^). The TRBF is *O*(*k*_2_ *n*) and the ERBF is *O*(*n*). So ITRN-RBF runs in *O*(*k*_1_ *Nlog*_2_ *N* + *n* ^3^ + *k*_2_ *n*) (based on TRBF) or *O*(*k*_1_ *Nlog*_2_ *N* + *n* ^3^) (based on ERBF).

We list the running times in Table 3. Specially, training RBF and mapping dataset are separated, so the extent to which RBFN maps the dataset fast are quite remarkable. We note that:

- In most applications, *n* < < *N*, so MDS and training RBFN run faster.
- If we get RBFN, mapping the dataset only costs *O*(*N*).
- ITRN-TRBF is slower than ITRN-ERBF because trained RBFN has iterative procedure. However, if we get RBFN, the mapping based TRBF is always faster than ERBF’s, because ERBF has larger number of center nodes in network.

**Table 3:** Running times (specified in seconds) for different methods.

Dataset	ITRN-ERBF		ITRN-TRBF	
	Training RBFN	Mapping	Training RBFN	Mapping
Swiss roll	24.3739	0.0626	47.5088	0.0031
AF	14.2524	0.2210	199.2901	0.2271
“2”	81.6783	2.8812	387.0357	0.3082
UMist face	5.7719	0.2558	588.1310	0.3176

Dataset	RBF-NDR	GNLP-NG	ISOMAP	L-ISOMAP	PCA
Swiss roll	18.2267	175.9537	11.7621	5.1544	0.0016
AF	41.6311	99.7312	1.1107	0.7062	0.0152
“2”	184.3450	106.8090	2.8570	1.4513	0.2179
UMist face	———	———	0.0443	0.0422	0.0293

## Discussion

The classical dimensionality reduction methods, such as PCA and MDS cannot disclose nonlinear embedded manifolds. ISOMAP and L-ISOMAP uses geodesic distantce to improve MDS, providing good performance. ITRN-RBF offers performance near that, but has a faster mapping speed and an ability to deal with new data.

For the dimensionality reduction methods based on TRN, OVI-NG can also not process nonlinear dataset because it uses Euclidean distances in the observation space. GNLP-NG makes improvements that are similar to ISOMAP’s. Both of OVI-NG and GNLP-NG cannot project new data online.

ITRN-RBF and RBF-NDR overcome these problems. They can project nonlinear data for using geodesic distances and can map new data because of RBFN. In this paper, we proposed two methods to obtain the RBFN. Each has distinct advantages and disadvantages. ERBF has only one parameter, its *spread*. Larger *spread* will generate more robust networks, but too large a *spread* will cause mathematical calculation problems. ERBF only calculates once without accumulating error, hence it is fast and exact. However, a large number of training patterns will result in a large-scale network. ITRN uses the vector quantization technique to decrease the number of training patterns, hence ERBF is the recommended approach to obtain an RBFN. The other method, TRBF, obtains a training RBFN, which requires a large number of adjustable parameters and calculation time.

Compared with RBF-NDR, ITRN-RBF has definitive results and high mapping quality. ITRN-RBF has good scalability with reasonable hardware costs. That is, if more effective methods for getting RBFN are adopted, better performance is obtained.

To sum up, the proposed ITRN-RBF that uses ITRN, which is suitable for geodesic distances because it builds a more appropriate topology relationship, does well with nonlinearly embedded manifolds, large amounts of data, and the online projection of new data. This method can be applied to a wide range of applications including visualization, feature extraction, and other applications.

## Supporting Information

S1 Dataset Randomly generated nodes dataset. (ZIP)

S2 Dataset Swiss roll dataset. (ZIP)

S3 Dataset AF dataset. (ZIP)

S4 Dataset Handwritten digit “2” dataset. (ZIP)

S5 Dataset UMist face dataset. (ZIP)
